# 2.5D printing of a yield-stress fluid

**DOI:** 10.1038/s41598-023-32007-7

**Published:** 2023-03-29

**Authors:** Simon Colanges, Jean-Noël Tourvieille, Pierre Lidon, Jacques Leng

**Affiliations:** 1 Univ. Bordeaux, Laboratory of the Future, CNRS, Solvay, 33600 Pessac, France; 2 Univ. Bordeaux, Centre de Recherche Paul-Pascal, CNRS, 33400 Talence, France; 3 Solvay Laboratory of the Future, 33600 Pessac, France

**Keywords:** Engineering, Soft materials

## Abstract

We report on direct ink writing of a model yield-stress fluid and focus on the printability of the first layer, the one in contact with the supporting substrate. We observe a diversity of deposition morphologies that depends on a limited set of operational parameters, mainly ink flow rate, substrate speed and writing density, and also on material properties (e.g., yield-stress). Among these morphologies, one of them does not depend on fluid properties (as long as the fluid displays some yield-stress) and consists of flat films whose thickness is controllable in a significant range, about $0.1{-}1$ mm, and tunable in real time during printing. We thus demonstrate the ability to print films with thickness gradients and prove that the printing fidelity is mainly due to a competition between yield-stress and capillarity.

## Introduction

While additive manufacturing is a flourishing field of research, innovation and, today, industrial development, there is a continuous effort to develop new techniques that enrich the field of possibilities; Direct Ink Writing (DIW) is one of them. It has been described exhaustively in a recent and impressive review^1^ that pointed the main force of DIW. It is an additive manufacturing technique that is not tied to a specific material, but rather a paradigmatic approach that relies on the rheological properties of formulated inks to achieve a simple goal: flow when necessary, solidify when not. The use of visco-elasto-plastic inks such as yield-stress fluids (YSF) addresses this constraint^2^ and opens up the world of physical chemistry to design formulations^3^. The range of printable materials is virtually unlimited, whether the materials are formed directly^4^ or embedded in a supporting gel^5^, and the process is quite inexpensive while being extremely versatile^1^.

In this paper, we address *a fundamental study of two-dimensional DIW* with a model yield-stress fluid, namely a Carbopol gel (which we also call ‘the gel’). It is a non-toxic, easy-to-formulate water-based YSF whose rheological features can be tuned directly by the amount and nature of packed microgel beads and which has been extensively studied^6–8^. Furthermore, being a crystal clear material, it can be tinted in order to carry out topographic imaging through colorimetry. Specifically, we looked at a very basic feature: how to take advantage of fluid yield-stress with DIW to print a continuous film in a single pass and dynamically modulate its morphology. This contrasts with most research papers that attempt to relate the rheological properties of the YSF to the ability to form a multilayer 3D structure that supports its weight and capillary forces^9–11^. Here we focus on the ‘single layer’ object.

Through careful analysis of the interplay between printing parameters such as flow rate, stage movement, etc., and the specific wetting of the YSF with the bed (the substrate receiving the fluid on the moving stage), we identify a regime where it is possible to write continuous films of gel whose thickness are controllable in a significant range only by the operational parameters, not the material parameters, as long as the fluid displays a yield-stress that can oppose either the weight of the film or capillarity due to edge menisci. Furthermore, we evidence that the film thickness can be changed in real time during printing through printing parameters, allowing films with thickness gradients to be designed in a single pass, *which we call here 2.5D printing*. We highlight that the vertical print resolution, i.e., the stiffness of the gradients, is controlled mainly by a competition between the yield-stress of the fluid and capillary forces. Gel interfaces with non-trivial surfaces can therefore be designed on the basis of ink rheology and may be of interest in many areas such as material or life sciences.

## Results

**Figure 1:**
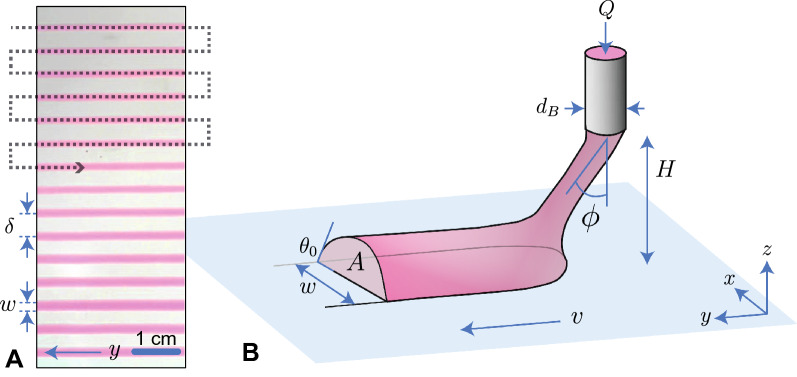
(**A**) The serpentine pathway highlighted with the dotted line permits us to evidence some specific writing regimes, here for instance the continuous regime where well defined straight lines are deposited and whose widths may be controlled during the deposition. (**B**) Realistic sketch of the deposition front which connects the fluid extruded from the injection nozzle to the deposited line on the bed. The writing parameters *Q* and *v* along with the wettability of the fluid onto the bed and its visco-elasto-plastic properties get combined to determine the morphology of the written line. The figure was created with Adobe Illustrator v27.1, see https://www.adobe.com/fr/products/illustrator.html.

The methodology is as follows: we have formulated a Carbopol YSF with a typical yield-stress ranging in $\sigma _y\approx 5$ Pa (see “Methods”), calibrated a colorimetric imaging technique to measure the thickness profiles of the dyed YSF deposited on a substrate (see “Methods”), and written lines and films of the YSF with a home-made DIW machine, only in 2D for the present study. Doing so, we have programmed the fluid deposition trajectory (Fig. 1A) as well as the writing parameters (Fig. 1B), mostly the bed linear velocity *v*, the height *H* of the printing nozzle, and the flow rate *Q* of fluid extrusion. Eventually, we analysed the deposit morphology such as shape and volume, contact angle, etc., against operational parameter. The most important characteristics that emerged from this approach were the (relative) stability of the process and the morphology of the deposits, of which the graded thickness films are, in our opinion, the most interesting.

### Stability

**Figure 2:**
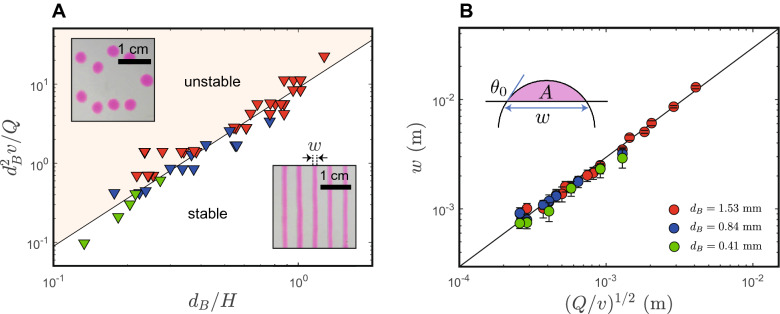
(**A**) Stability map of ‘printability’ separating continuous/stable from discontinuous/unstable printing (see inserts). The solid line is an guide scaling with a power 2; colours as in (**B**). (**B**) Footprint *w* of the deposit upon stable printing as a function $Q/v\equiv A$, the volume per unit of length of deposit. The solid line is a fit, see text, showing a linear relationship between footprint *w* and $(Q/v)^{1/2}$; the prefactor ($3.0\pm 0.1$) fits with trigonometry. $d_B$ is the internal diameter of the nozzle.

Any DIW process requires a matching of the fluid rheology and the deposition process. For example, sometimes during the printing process the filament of fluid breaks up into separate droplets (see top-left insert in Fig. 2A) rather than remaining in a continuous filament as desired. Literature data is scarce about this critical issue^12,13^, although there is some empirical expertise, especially in the field of bioprinting, that suggests that the nozzle height should be at most $\approx 1.5$ times the nozzle diameter. It is also common sense that the velocity of the fluid flowing out of the nozzle should roughly match the bed velocity to ensure material continuity. We show below that stability results from a complex interplay between the printing parameters, the surface state of the bed, that of the nozzle, and the rheological properties of the fluid.

When the process is stable, the gel lines can be printed continuously indeed and form a cylindrical cap due to partial wetting of the gel on the glass (Fig. 1A,B) and the printed line remains connected to the nozzle by a slanted filament. The surface state of the bed along with the dynamic arrest of the YSF upon spreading determine the final contact angle^14^ of the cylindrical cap, which we will comment on later. However, there are conditions, such as too rapid a deposition, too low a flow rate, or a nozzle that is too high, that lead to jet breakage; the inner diameter of the nozzle also plays an indirect role.

The respective roles of the printing parameters were investigated using the following basic protocol: the gel was deposited along a serpentine trajectory (Fig. 1A) at given *Q*, *v*, and $d_B$; along each straight line of the serpentine, the nozzle was raised from a low reference height to a higher position and then lowered again before the turn (see video provided as supplementary material). This allowed us to bypass jet instability at the level of the half-turn related to ill-controlled wetting and meniscus anchoring (see video provided as supplementary material). Again using video, we have shown that there is a critical height $H_C$ above which the jet breaks up. Iterations of the protocol over different *Q*, *v*, and $d_B$ led to an overview of the stability of the process and we were thus able to collect the data points at the onset of the instability.

In Fig. 2A we plot the data points at the onset of the instability as a function of a set of reduced parameters. This figure shows that these data points define the limit between two domains, continuous *vs* unstable printing, and the boundary between these two domains scales roughly like $d_B^2v/Q \sim (d_B/H_C)^2$ (solid line, Fig. 2). The choice of these dimensionless groups is guided on the one hand by the comparison of the bed velocity *v* with the fluid velocity at the nozzle $Q/d_B^2$, and on the other hand by a geometric factor $H/d_B$ which captures the aspect ratio of the filament. Experimentally, the stability criterion therefore reads $H^2 < \beta Q/v$ with $\beta \approx 9$ for this gel and where the diameter of the nozzle $d_B$ does not seem to be directly relevant. However, the nozzle diameter $d_B$ does influence the area of operation, since larger nozzles permit access to greater printing heights, see the sequential overlap of colour symbols in Fig. 2. In addition, the wetting properties of the fluid on the nozzle are important in defining the boundary conditions. Here, the nozzle has non-wetting conditions, which leads to defining the inner diameter as its typical length scale.

These observations suggest that stability of the process is related to the aspect ratio of the jet, and that its break-up could be caused by a Rayleigh-Plateau type instability. The exact analysis of this instability is made difficult mostly because the geometry of the filament is far from being canonical and is out of the scope of the present work. However, some important factors become clear when we question what determines its geometry: upper boundary conditions are fixed at the level of the nozzle (non wetting and average velocity $\sim Q/d_B^2$); the yield-stress fluid which has undergone a partial fluidisation close to the inner walls of the nozzle soon recovers an elastic behaviour and eventually touches the bed (even though full recovery can take tens of seconds, see the section on rheology in the supplementary material). There, the YSF spreads upon wetting and spreading stops when the yield-stress balances capillarity; the role of the (dynamic) yield-stress is thus of prime importance as it stabilizes the printed pattern.

The filament that connects the nozzle to the printed line basically consists of a piece of gel that flows and which may be also elastically deformed under the strain due to the displacement of the bed at constant velocity. The elastic stress it may store scales like $\sim G'\tan \phi$ where $\phi$ is the angle the filament adopts with respect to the vertical *z* axis (Fig. 1B) and where $G'$ is the elastic modulus of the gel. This elastic stress is limited by the yield-stress and consequently, above this threshold, a local fluidisation of the gel is likely to happen where it connects with the printed line. The angle $\phi$ thus scales like $\sim \arctan {(\sigma _y/G')}$. In this naive description, the angle of the filament is solely due to material properties and upon raising up the nozzle (*H*), the angle remains unchanged but the aspect ratio of the filament (diameter/length) drops and a Rayleigh-Plateau instability may develop, which can also be controlled by the mechanical properties of the gel^15^. Note however that this hypothesis is not universal^16^ and is also highly dependent on the elongational properties of the fluid, if any^17^.

### Footprint

Single layer printing is essential as it forms the basis of the most common paradigm in additive manufacturing, namely layer by layer printing. The first layer, however, is specific as it involves the interaction of the extrudate with the bed. Here we characterise this interaction and then try to take full advantage of it.

Figure 2B summarizes the relationship between printing parameters and morphological features of the printed lines in the case of stable printing. We demonstrate that lines have smooth and parabolic sectional profiles (Fig. 7B) which are analysed using by colorimetry to extract transverse height profiles. We obtain the footprint of the line *w*, that is its size at the level of the substrate, and *A* the area under the profile. We also measured the contact angle $\theta _0$; the latter depends on the exact amount of deposited material through *Q*/*v* but tends to an asymptotic value $\theta _0 \approx (30 \pm 3)^{\circ }$ in close agreement with recent results on the same gel^14^. Of course, this contact angle depends on the surface properties of the bed, here a glass substrate with no specific treatment; for a series of limited and dedicated tests, we made it hydrophobic through silanization and confirmed the impact of a higher contact angle on a smaller footprint.

The outcome of this systematic investigation is quite direct. The footprint *w* depends only on the printing parameter *Q*/*v* and the contact angle of the gel on the bed, not on the nozzle diameter (Fig. 2B). Notice that it could be different with fluids that die-swell at the extrusion nozzle but this is not the case of the Carbopol we used here. *Q*/*v* represents the volume deposited per unit of length of the line and is thus the area under the profile: $Q/v\equiv A$, Fig. 2B. The way this material spreads depends both on the fluid and the substrate, and for a YSF such as Carbopol, spreading dynamically stops at some specific contact angle $\theta _0$ thus defining the related footprint *w*. There is a trigonometric relationship $w = \Gamma (Q/v)^{1/2}$ where $\Gamma$ depends only on $\theta _0$. Assuming a partial cylindrical cap as sketched in Fig. 2B, we obtain $\Gamma (\theta _0) = 2\sqrt{2} \sin {\theta _0}/\sqrt{(2\theta _0 - \sin (2\theta _0))}$ which translates into $\Gamma = 3.3\pm 0.2$ using the asymptotic value of $\theta _0$ given above. It matches perfectly our observations obtained with a fit with $\Gamma _{\text {exp.}} = 3.0\pm 0.3$. There is therefore an non-ambiguous and predictable relationship between *w* and *Q*/*v*.

### Overlap

The footprint of the printed gel line is thus directly tunable through the printing parameter *Q*/*v*. Adjacent lines will overlap if their separating distance $\delta$ (Fig. 1A) is smaller than the footprint *w* and thus the ratio $\delta /w$ is the dimensionless criterion describing overlap and depending this ratio, we observe several morphologies of the deposit.

**Figure 3:**
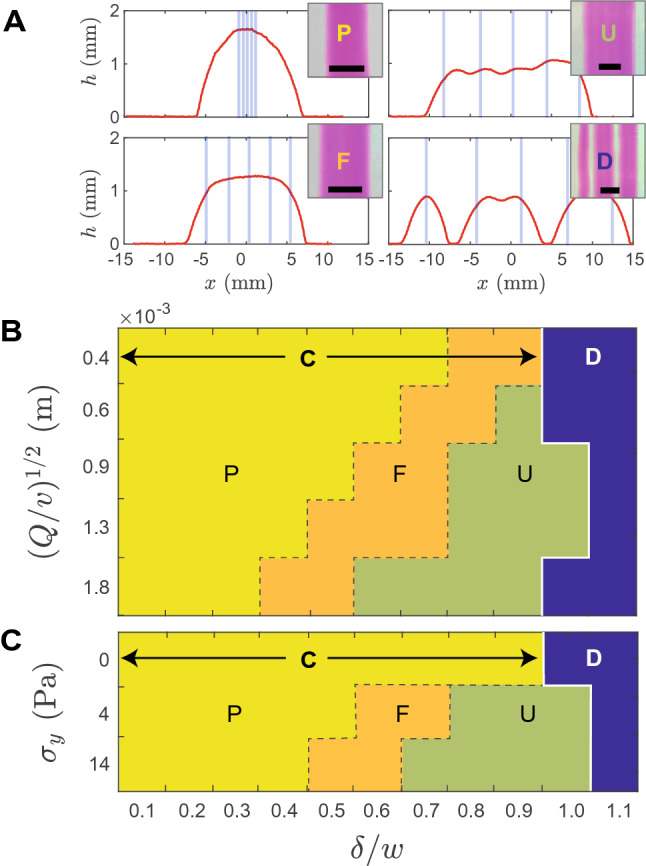
(**A**) Depending the overlap ratio $\delta /w$ of printed lines (here 5 lines), different morphologies of the deposit are observed: discontinuous (D) or continuous, with undulating (U), flat (F) or parabolic (P) profiles ($Q/v=8.3\,10^{-7}$ m$^2$, and $\delta /w=1.1,\,0.8,\,0.5$ and 0.1 respectively. The blue vertical lines indicate the position of the print path). The cartography of these morphologies depends on the on the overlap ratio $\delta /w$ and printing parameters *Q*/*v* (fixed yield-stress $\sigma _y = 4$ Pa) (**B**) and also on the yield-stress $\sigma _y$ of the fluid (fixed $Q/v=8.3\,10^{-7}$ m$^2$) (**C**).

Figure 3 describes the consequence of a tighter and tighter overlap (decreasing value of $\delta /w$) on the shape of *five *adjacent lines. Starting from $\delta /w \approx 1$, we observe that the line are disconnected, as expected, even though some overlap may occur (pattern D, Fig. 3A). As soon as $\delta /w < 1$, a continuous pattern is printed either with undulations and wrinkles when $\delta /w \lesssim 1$ (pattern U, Fig. 3A), flat (pattern F, Fig. 3A), or parabolic (pattern P, Fig. 3A) when $\delta /w \ll 1$.

At a fixed yield-stress, the boundaries separating these morphologies depend on the actual printing parameters and tend to fade away at low *Q*/*v* (Fig. 3B). At a given printing parameter, the yield-stress of the fluid also impacts the cartography and obviously, when the yield-stress vanishes, the different regimes disappear but increasing the yield-stress also tends to shift the boundaries between regimes (Fig. 3C). While the interplay between capillarity and yield-stress at the origin of this behaviour will be discussed later, the F and P schemes are particularly interesting; the former opens up the possibility of printing a flat deposit, while the latter adds up several deposits to create an equivalent deposit with a much larger volume while retaining the natural profile of a single line.

### Continuous films with controllable thickness

**Figure 4:**
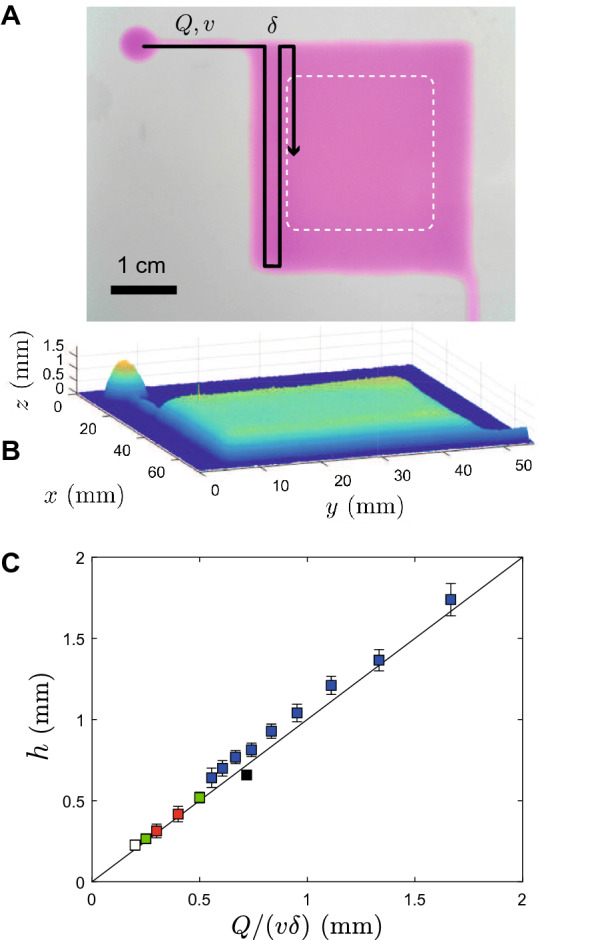
(**A**,**B**) A continuous flat film can be printed upon proper overlap of the lines; the thickness of the deposit is measured by averaging the data points collected in the white dotted area in (**A**), and depends on a simple combination of the printing parameters *Q*, *v*, and $\delta$ (**C**, different colours of symbols correspond to different sets of printing parameters). The straight line has a slope of 1.

We are now focusing on F and P regimes, i.e., highly overlapping printing regimes that have allowed us to form continuous deposits without process memory, such as surface wrinkles. Figure 4A shows such a continuous and flat film of gel. Colorimetry was used to obtain the thickness profile of the deposit, Fig. 4B, and the thickness *h* was measured in the central region of the layer to exclude any bias due to edge effects (white dashed square, Fig. 4A). The calculation of *h* is thus an average over $\approx 5\times 10^4$ point measurements leading to a standard deviation of about 5% relative to *h*. These films thus fairly homogeneous and the thicknesses obtained here range in $h\approx 0.2-2$ mm depending on the chosen set of printing parameters *Q*, *v*, and $\delta$, see Fig. 4C. More precisely, as *Q*/*v* is the linear volume density along a line and $1/\delta$ the spatial frequency of lines, $Q/(v\delta )$ is the volume deposited per unit of area, homogeneous to a length that matches exactly $h\equiv Q/(v\delta )$, as demonstrated in Fig. 4C (straight line with slope 1, no fit).

The film thickness *h* is thus entirely controllable via the printing parameters (*Q*/*v*) over a significant range. Consequently, *h* does not depend on the fluid material properties, as long as its flow and mechanical properties allow us to reach a continuous and stable regime (no memory effect, P and F regimes). However, the boundaries of the various domains (P, U, F and D) *do* depend on the fluid material properties.

It should also be noted that a YSF allows us to print a flat film at a thickness significantly different from the capillary length (of water for example as the gel contains only $\approx 0.3\%$ of polymer in weight). Such a configuration remains mechanically stable only through the action of the gel yield stress and we believe that we can take advantage of this stability to adjust the printing parameters in real time to adjust the local film thickness.

### Films with gradients of thickness

**Figure 5:**
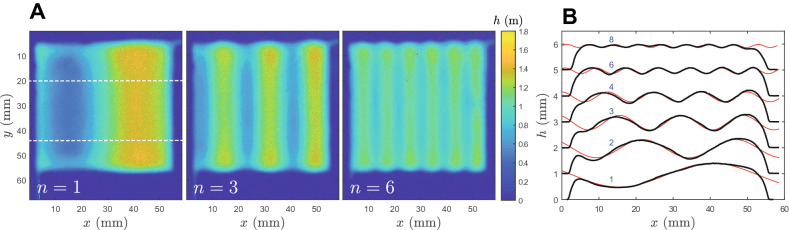
(**A**) Thickness gradient were obtained with a variation of overlap distance $\delta$ at constant flow rate and deposition velocity: here, a sinusoidal pattern has been imposed with a constant average height (1 mm) and fluctuation magnitude (0.5 mm); the number of periods $\lambda$ can be varied along the writing distance *L* and is characterized by $n=L/\lambda$; the dashed lines show the zone where the profiles have been averaged to obtain part (**B**) where the index *n* is given for each curve (which are systematically shifted by 1 mm for the sake of clarity).

As $h\equiv Q/(v\delta )$, it seems obvious that the time/space adjustment of any of the related printing parameters should open the possibility of creating a time/space dependent thickness. We chose to keep the flow rate constant throughout the process because it is known that there is some time delay in the fluidic system^18^ when changing *Q* (due to syringe pumps, syringes, tubes, etc.). In this context, flow control by pressure could be advantageous.

For simplicity, we kept the bed velocity constant and changed only the overlap value in order to encode a canonical shape, namely a sinusoid. We therefore sought to print a film of spatial extent $L\times L$ (here $L = 5$ cm) with a thickness varying in a single direction *x* ($\in [0-L]$): $h(x) = h_0\left[ 1 + \alpha \sin {(2\pi nx/L)} \right]$ where $h_0$ is the mean thickness we fixed at around 1 mm, $\alpha <1$ the magnitude of gradients relative to $h_0$, $2\pi n/L$ the wavenumber of the thickness oscillations where we chose $n\in [1-8]$. $\alpha$ can be considered as a parameter describing the roughness of the film.

It is important to note that not all $\alpha$ values are accessible due to the printability characteristics of the gel described above: there is a trade-off between filament stability and film morphology that selects the possible ranges of printing parameters, which we exemplify below with real values.

The choice of a set of printing parameters *Q* and *v* and a nominal thickness $h_0$ automatically selects the nominal writing density $1/\delta _0$. Then, the choice of a specific roughness $\alpha$ leads to a locally varying thickness that will eventually have an impact on the printing regime and the stability limit. The combination of all these constraints allows us to define the range of possible parameters.

For example, with a set of selected values (say $Q=0.5$ mL/min, $v=30$ mm/s $\rightarrow$ $(Q/v)^{1/2} \approx 5.3\,10^{-4}$ m), the nominal footprint is $w_0\approx 1.7$ mm (Fig. 2B) and the nominal overlap required to reach $h_0=1$ mm is $\delta _0 \approx 280$ μm (Fig. 4C leading to $\delta _0/w_0\approx 0.17$). Beside, in order to remain in the P regime, the range of overlap accessible is $\delta /w_0 \approx [0.1{-}0.6]$ (Fig. 3B) which allows us to delineate the maximum gradients to be reached via $\delta \approx [0.17{-}1]$ mm and thus $h\approx [0.27{-}1.6]$ mm. The maximum magnitude of gradients is $\approx \text {min}(|h-h_0|)$, thus $\approx 0.6$ mm limiting $\alpha \lesssim 0.6$. This range of accessible gradients can be enlarged if we accept to work in the F regime ($\delta /w_0 \lesssim 0.8 \rightarrow \alpha \approx 0.2$) or if we explore tighter overlap (e.g., $\delta = 0.05 \rightarrow \alpha \approx 3.3$). However, in the latter case, such a very strong overlap causes a supposedly very thick deposit that may interfere with the printing nozzle. As we do not wish the nozzle to be immersed in the gel, it seems possible to raise its level so that we always ensure $h<H$ yet we also require stable printing (Fig. 2A) which reads for this gels $H^2 \lesssim 9 Q/v$, that is $H \lesssim 1.6$ mm, which sets the upper limit of the deposit we can print in one pass; higher values of *H* are accessible but require to tune *Q* and *v* to explore a different region in the stability map (Fig. 2). We thus recognise that the interplay between stability and deposit morphology defines and constrains a window of printing parameters, which are also material-dependent.

Yet, the approach is conclusive: Fig. 5A shows three thickness maps of sinusoidal deposits printed in one pass with increasing wavenumbers. While these maps convincingly illustrate the ability to tune the thickness in space, a more precise analysis on the thickness averaged over a significant portion of the deposit shows a discrepancy between programmed and printed gradients. Here, the set points were $h_0=1$ mm, $\alpha = 0.5$, and $n\in [1{\cdots }4,6,8]$ and Fig. 5B shows the averaged profiles along the *x* direction as well as a sinusoidal fit. In all cases, whatever the wavenumber, the period of printed thickness gradients matches perfectly the requirement but the magnitude of the gradients diminishes as the number of periods increases.

## Discussion

**Figure 6:**
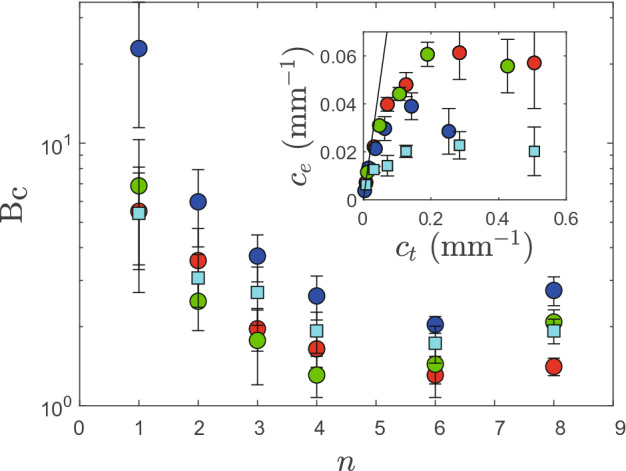
Bingham capillary number B$_\text {c}$ as a function of the number of printed oscillations for several magnitudes of gradients and yield-stresses ($\sigma _y \approx 5$ Pa for blue, red, and green circles with $\alpha =0.25, \, 0.5$ and 0.6 respectively, and $\sigma _y \approx 2$ Pa for the cyan square with $\alpha = 0.5$). Insert: Experimental final curvature $c_e$ against the prescribed one $c_t$; the solid line has a slope of unity.

We have described above the possibility of printing films whose thickness can be directly modified by the operational parameters of the printing process. It opens the way to the variation of thickness in space and thus to the programming of thickness gradients. We have explored this perspective here with a ‘weak’ YSF ($\sigma _y \lesssim 15$ Pa) and demonstrated an important result: if gradient films are indeed printable, there is a limit to the magnitude of the gradients that can be printed compared to those programmed. Simple arguments opposing capillarity to yield-stress can explain this result.

The height profiles *h*(*x*) of the gradient films have been extracted (Fig. 5B) and their curvature can be calculated: $c(x) = h''(x)/[1+h'(x)^2]^{3/2}$, which we have done on the basis of the fit to reduce noise. We have kept the maximum value of the experimental curvature $c_e$ and calculated the theoretical curvature which scales as $c_t = \alpha h_0 (2\pi n/L)^2$. The insert of Fig. 6 clearly shows that experimental curvature deviates from the programmed one and then plateaus. We also observe that this effect is more pronounced with a weaker gel (cyan squares).

We believe that during the printing process, if strong curvatures are created, they relax under capillary forces until the gel is blocked due to its dynamic yield stress. Based on the curvature calculation, we can estimate the Laplace forces at the air/gel interface $\sigma _c = \gamma c_e$ where $\gamma \approx 60$ mN m$^{-1}$ is the air/gel surface tension^14^ and compare them to the gel yield-stress. In doing so, we define the Bingham capillary number B$_{\text {c}} = \sigma _y/\sigma _c$, a dimensionless number which compares both effects. Fig. 6 shows that upon increasing the number of oscillations, and therefore curvature, the Bingham number decreases from B$_{\text {c}}\gg 1$ to B$_{\text {c}}\approx 2$ indeed suggesting that Laplace forces are responsible for reducing the curvature of films with strong height gradients. It is worth mentioning that such a description is supported by recent results on YSF drop coalescence^19^ but we can also question the role of the elasticity of the gel $G'$ which may also limit the coalescence of solid filaments surrounded by a fluidized YSF, as highlighted in Ref.^20^.

On the basis of the sinusoidal shape we programmed, we calculate that $\sigma _c \sim \sigma _c^0\alpha n^2$ where $\sigma _c^0 = 4 \pi ^2 \gamma (h_0/L^2)$ appears as the basic value for the Laplace force acting on sinusoidal interfaces with specific dimensions, while modulations of thickness then involve the magnitude and wavenumber. If we assume that high-fidelity printing can be achieved when B$_{\text {c}}\gg 1$, we get a requirement on the gel yield-stress: $\sigma _y \gg \sigma _c^0\alpha n^2$. Here, with $h_0 = 1$ mm and $L=5$ cm, $\sigma _c^0 \approx 1$ Pa and shallow profiles (e.g., $\alpha = 0.1$) at low wavenumbers ($n=1$) lead to $\sigma _y \gg 0.1$ Pa, and are thus easily accessible while high-frequency modulations are more demanding, e.g., $n=10$ requires $\sigma _y \gg 10$ Pa. Large magnitude gradients are even more stringent with for instance $\sigma _y \gg 100$ Pa for $\alpha = 1,\,n=10$. Note that such a range of yield-stresses ($\sigma _y = 1{-}10^3$ Pa) is compatible with formulations encountered in direct-ink writing^3,9,21^.

## Methods

### The yield-stress fluid

The yield-stress fluid (YSF) we use is Carbopol (ETD 2050 from Lubrizol) which is a suspension of packed microgels. It is dyed with Rhodamine B (from Merck). The preparation protocol is the following: 300 mL of ultra-pure water are introduced in a beaker which is heated at $\approx 50$ °C. 0.9 g of Carbopol is slowly introduced while magnetically stirring the solution. Once the Carbopol powder is completely dissolved in the solution ($\approx 1$ h), the latter is left to stand for 30 min at room temperature. Gelation of the fluid is then triggered by adjusting the pH from 3 to 7 (controlled with a pH-meter) using drop-wise addition of a 10 M sodium hydroxide solution under mechanical stirring. 39 mL of a stock solution of rhodamine B (at 2 mM in ultra-pure water) are eventually added and thoroughly stirred until homogeneous dying the fluid.

The final stage consists in mixing the above-mentioned fluid with water in order to tune the yield-stress of the fluid. Here, we use 250 mL of the dyed-Carbopol with 250 mL of water which leads to a mass percentage of Carbopol of 0.13% and a rhodamine concentration of 0.12 mM, which is the YSF we used for most of the experiments reported here.

For one set of experiments, we used Carbopol preparations at 0.04% and 0.26% mass fractions corresponding to $\sigma _y \approx 0$ Pa and $\sigma _y \approx 12$ Pa respectively, prepared directly to final concentration and not by dilution of a concatenated stock solution.

### DIW machine

The printing machine includes a 3-axis platform (Cetoni GmbH), flow control by Nemesys syringe pumps (Cetoni GmbH) and imaging. The bed moves horizontally along *x* and *y* axes (velocity range $v=0.1{-}180$ mm s$^{-1}$, position accuracy $\approx 1$ μm) while the printing nozzle is mounted on the *z* axis. The latter consists of a luer-lock based needle tip made of passivated stainless steel (Nordson EFD) with controllable diameter connected to a 10 mL glass syringe (VWR) via a 1/8″ (outer diameter) Perfluoroalkoxy tubing and flangless fitting solutions (nuts, ferrules, and luer lock adaptors from Idex). As a bed, we used a $10\times 10$ cm, 3 mm-thick glass substrate to take advantage of transparency in order to image printed patterns in transmission mode straight after printing. To do so, the bed is sent to a registered position inside the printing housing and illuminated by a flat LED back light panel and imaged using a Microsoft LifeCAM Studio webcam. All commands (displacement, flow, images) are integrated into a Matlab code.

### Colorimetry calibration

**Figure 7:**
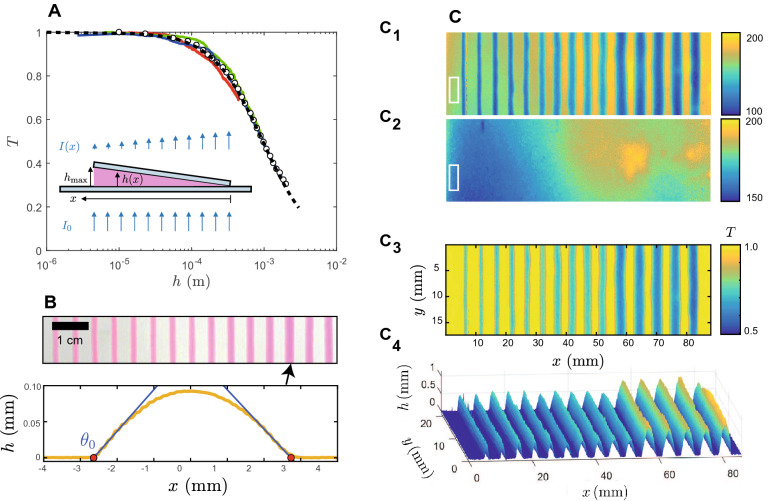
Colorimetric calibration. (**A**) Transmission of a dyed gel measured against thickness in a wedge geometry where $h_{\text {max}}$ is known. The coloured lines correspond to several $h_{\text {max}}$, the open symbol show the average of these data, and the black dashed line an empirical fit ${\mathscr {F}}(h)$. (**B**) Linear deposits are obtained for several *Q*/*v* and the height profiles are measured using ${\mathscr {F}}$; an example of profile is shown, from which the deposited area *A* and the contact $\theta _0$ angle can be measured. (**C**) Converting an image (C1) into a topographic measurement (C4) through the acquisition of a reference (C2), giving the transmission *T* (C3) and the height profiles (C4).

The colorimetric calibration is a multi-step process that permits us to convert the colour level of an image from a simple webcam into a thickness. Even though not as quantitative as a spectroscopic measurement, it nevertheless yields fruitful information. Before describing the procedure, we notice that we impose manually some settings of the camera, such as the focus length, the gain, the white balance correction, etc. On the other hand, some slight variation of the lighting conditions are unavoidable and cannot be controlled, and are taken into account. The calibration procedure is as follows:

- we first set up a corner-like geometry (see insert of Fig. 7A, top) in between two glass slides in order to acquire an image of the dyed gel with a continuously varying thickness and we also acquire the same image for a blank gel (same gel, no dye). The division of the two images yields some transmittance map *T* that leads to an initial law linking transmittance and thickness: $T = {\mathscr {F}}(h)$ (Fig. 7A). Here, we take advantage of the strong absorption of rhodamine to focus on its complementary color, i.e., green, and limit the analysis to the green channel of the RGB camera. In Fig. 7A, the coloured lines correspond to several corner geometries (3 different $h_{\text {max}}$ which we know exactly) that show both some consistency and the experimental error; the open symbols correspond to an average over all the measurements. Eventually, we devise an empirical fit: ${\mathscr {F}}(h) = 1/(1+\beta h^{\alpha })$, see dashed line of Fig. 7A;
- then, we print several lines (Fig. 7B) for a large set of the parameter *Q*/*v* and measure the thickness profiles using the initial calibration ${\mathscr {F}}(h)$; it also leads the deposited area *A* which must satisfy volume conservation (assuming no evaporation/drying, which we have checked) and thus should be a linear function of the deposition parameter $A \equiv Q/v$. We find this is not perfectly true (not shown) which we attribute to the optical difference between calibration and measurement: indeed, there is a glass coverslip on top of the corner-like geometry whereas the written line has a free interface with air, which is likely to change slightly the transmitted intensity; we thus operate a fine tuning in order to comply with volume conservation. ${\mathscr {F}}\rightarrow \tilde{{\mathscr {F}}}= 1/(1+{\tilde{\beta }} h^{\alpha })$ permits us to get a near-quantitative analysis of the thickness of any deposit owing its thickness does lead to a transmittance smaller than $\approx 0.2$. Eventually and for the sake of simplicity, we attribute a 10% systematic relative error to thickness measurement.

The outcome is a semi-quantitative procedure that permits us to transform an image acquired with a low-cost webcam into a thickness map, as illustrated in Fig. 7C: we acquire an image of some deposited pattern (C1) and a blank image (C2, including the glass substrate). Some slight illumination variations that may occur between the acquisition of the two images due to the fluctuations of the backlight are taken into account by defining a normalization zone, the white rectangle in Fig. 7C1,C2. Owing this normalisation, the division of the two images gives the transmittance map *T*(*x*, *y*), Fig. 7C3. Then, we apply pixel by pixel the calibration curve $\tilde{{\mathscr {F}}}$ in order to obtain the thickness map *h*(*x*, *y*), Fig. 7C4, including a spatial calibration.

## Conclusion

Continuous films with gradients of thickness may find interest in many fields such as optics, biology, material science, micro-technology, etc., and have been fabricated using for instance flow coating^22^ or grey-level lithography^23^. Here, we present an alternative method, 2.5D printing, that relies on both mechanical properties of yield-stress fluids and on the ability to adjust the process parameters of DIW in real time. It is based on fluids as does flow coating for example yet makes it possible virtually any type of gradient as grey-level lithography. So far, the range of thicknesses discussed is $0.1{-}1$ mm, which can be extended, and through experiments and a dimensional analysis, we suggest that the higher the yield-stress of the YSF, the steeper gradients are printable at high fidelity.

It is interesting to recall, as in ref.^1^, that rheology is both the foundation and stumbling block in the formulation of inks for DIW. While we believe that the high yield-stress values raise interesting questions about gradient printing, there are other problems that deserve more comprehensive studies: the flow in the nozzle, whether it is pressure or flow rate controlled, conditions the extent of the YSF fluidisation, mostly close to the wall of the nozzle, and it is not directly related to bulk rheology^24^; the kinetics of stress recovery immediately after the extrusion nozzle is important in determining the stability of the jet and the state of the fluid during deposition; partial coalescence of YSF filaments during deposition depends on geometry and the interplay between yield-stress^19^ and elasticity^20^ which will impede the print fidelity we can expect.

Finally, due to the range of materials that can be formulated as YSFs^1,3^, we expect that DIW with thickness (and possibly composition^25,26^) gradients will again enrich the range of applications. For instance, there is a link that can be made with non-planar 3D printing^27^ as the latter technique makes use of a curved substrate to generate smooth surfaces. In the context of 2.5D printing, there is no doubt that we can stack layers with adjustable thicknesses to generate a local curvature to be used as a substrate for non-planar 3D printing, for example. However, there is still a lot to be done to get the most out of 2.5D printing, especially with regard to the formulation of the YSF and the final morphology of the deposit.

## Supplementary Information

Supplementary Video 1.

Supplementary Video 2.

Supplementary Video 3.

Supplementary Information.

## Data Availability

The datasets used and/or analysed during the current study available from the corresponding author on reasonable request.
