# Supplementary material for: 2.5D printing of a yield-stress fluid
Source: Sci Rep. 2023 Mar 29;13:5155. doi: 10.1038/s41598-023-32007-7 (PMC10060369; doi:10.1038/s41598-023-32007-7)
Supplement: Supplementary file 4 — Supplementary Information. [file 41598_2023_32007_MOESM4_ESM.pdf]

Supplementary Information
2.5D Printing of a Yield-Stress Fluid

Simon Colanges[1,2], Jean-Noël Tourvieille[1,3], Pierre Lidon[1], Jacques Leng[1,*]

[1]Univ. Bordeaux, Laboratory of the Future, CNRS, Solvay, Pessac, 33600, France
[2]Univ. Bordeaux, CRPP, CNRS, Talence, 33400, France
[3]Solvay Laboratory of the Future, Pessac, 33600, France
*jacques.leng@cnrs.fr

1 Rheological Properties of the yield-stress Fluid

Carbopol is a yield-stress fluid with many different grades which, although they all behave in the same way, have subtle differences mainly concerning memory effects and wetting. This is due to the size and chemistry of the packed microgels. The one we used here, ETD2050 for 'Easy To Disperse', has differences between static and dynamic yield-stresses and a specific protocol is needed to properly characterise its rheological properties.

Here, we focused exclusively on the dynamic yield-stress which is described by a plateau at vanishingly-small shear rate as the the shear stress is decreased, see Fig. 1, left. In order to prepare the Carbopol prior to measurement, we pre-sheared it at $\dot{\gamma} = 100\ \mathrm{s}^{-1}$ for 1 minute and then applied a decreasing ramp of shear stress using a stress-controlled rheometer (Kinexus Ultra +, Malvern). We eluded the study of the static yield-stress as we believe it does not address the issues we described in the paper.

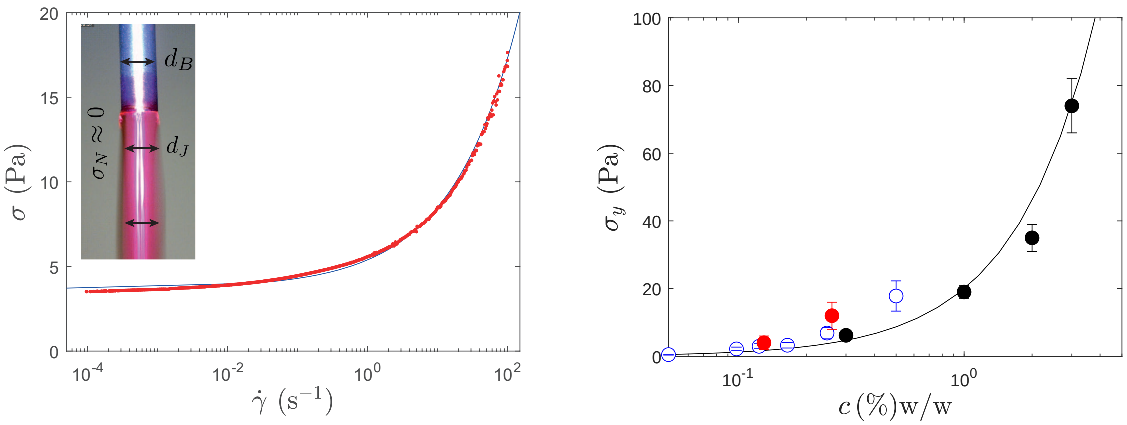

Supplementary figure 1: Left: Flow curve of the yield-stress fluid where the red symbols show the measurements and the solid line a fit with a Herschel-Bulkley model leading to a yield stress $\sigma_y \approx 4$ Pa, see text for the detail of the protocol; insert: close up of the extrusion needle showing that the diameter d_J of the jet is very similar to the one of the nozzle d_B (the edge of the jet is slightly blurred as a result of the image acquisition conditions). Right: Yield-stress obtained in the present study (red symbols) and from literature (blue symbols [1], black symbols [2]) as a function of mass fraction of Carbopol (ETD2050).

We analysed the flow curve using a typical HerschelBulkley fit, see Fig. 1 left, with $\sigma = \sigma_y + k\dot{\gamma}^{\alpha}$. For the example shown here, an ETD2050 Carbopol prepared at $c = 1.3\%$ w/w, we obtained a yield-stress $\sigma_y = (4 \pm 1)$ Pa, a consistency index $k \approx 1.7$ and the flow index $\alpha \approx 0.45$. We were unable to identify any contribution from normal forces, which also explains the absence of die swelling, as suggested by the insert in Fig. 1 showing a close-up of the extrusion needle used for printing.

For this precise material, we also measured the elasticity after a pre-shear at $\dot{\gamma} = 100$ s^{-1} for 1 minute followed by a 1 minute recovery time (data not shown). We identified it was sufficient for a full recovery of the elastic behaviour. Also, the linear regime at 1 Hz is limited to a few Pa and measurements within this linear regime of the elastic moduli in the linear regime led to $G' = (6 \pm 2)$ Pa and $G'' = (2.5 \pm 1.5)$ Pa for $f < 1$ Hz.

The values we obtained in this study are compared with the data in the literature [1, 2]. The right-hand side of Fig. 1 shows that our data (red symbols) are quite compatible with the literature data, although there are differences which we explain by different preparation protocols which are known to lead to different sizes of microgel aggregates, and thus possibly to different values of yield-stresses.

2 Legends of Supplementary Videos

We provide three videos that illustrate the possible instability of the printing process, as described in Fig. 2A of the main text.

Supplementary Video 1: The video entitled 'Limit of Stability' shows the result of the best protocol we have found for extracting the values of the printing parameters for which the process becomes unstable (here Q, v, d_B, and H). It consists in progressively increasing the height of the nozzle along a linear part of the print path until the jet breaks. When the jet breaks up, we obtain a set of printing parameters that describe the stability limit (Fig. 2A of the main text). Crucially, we found that we had to decrease the nozzle height before the U-shaped part of the serpentine path because this U can induce instability.

Supplementary Video 2: The U-turn instability can be seen in the second video entitled 'Instability after the U-turn' where the too high nozzle position and the specific anchoring of the meniscus at this position induce instability.

Supplementary Video 3: The video entitled 'Stick-slip instability' shows that a specific sliding movement of the meniscus (called here stick-slip) sometimes occurs at the same time as the filament rupture.

3 Data Availability

The datasets used and/or analysed during the current study available from the corresponding author on reasonable request.

References

[1] Thomas Brunet, Simon Raffy, Benoit Mascaro, Jacques Leng, Régis Wunenburger, Olivier Mondain-Monval, Olivier Poncelet, and Christophe Aristégui. Sharp acoustic multipolar-resonances in highly monodisperse emulsions. *Applied Physics Letters*, 101(1):011913, 2012.

[2] Grégoire Martouzet, Loren Jørgensen, Yoann Pelet, Anne-Laure Biance, and Catherine Barentin. Dynamic arrest during the spreading of a yield stress fluid drop. *Physical Review Fluids*, 6(4):044006, 2021.
